# Experimental Adaptation of the Yellow Fever Virus to the Mosquito *Aedes albopictus* and Potential risk of urban epidemics in Brazil, South America

**DOI:** 10.1038/s41598-018-32198-4

**Published:** 2018-09-25

**Authors:** Fadila Amraoui, Adrien Pain, Géraldine Piorkowski, Marie Vazeille, Dinair Couto-Lima, Xavier de Lamballerie, Ricardo Lourenço-de-Oliveira, Anna-Bella Failloux

**Affiliations:** 1 Department of Virology, Institut Pasteur, Arboviruses and Insect Vectors Unit, Paris, France; 2 Institut Pasteur - Bioinformatics and Biostatistics Hub – C3BI, USR, 3756 IP CNRS Paris, France; 3 Aix Marseille Université, IRD French Institute of Research for Development, EHESP French School of Public Health, EPV UMR_D 190 ‘Emergence des Pathologies Virales’, Marseille, France; 4 IHU Méditerranée Infection, APHM Public Hospitals of Marseille, Marseille, France; 5 Instituto Oswaldo Cruz - Fiocruz, Laboratório de Mosquitos Transmissores de Hematozoários, Rio de Janeiro, Brazil

## Abstract

Despite the availability of an efficient vaccine, Yellow fever (YF), a viral disease transmitted by mosquitoes, is still a threat. In Brazil, the yellow fever virus (YFV) has been restricted to a jungle cycle for more than 70 years. However, YFV has recently invaded populated cities in the Southeast such as Rio de Janeiro where the opportunistic mosquito *Aedes albopictus* is well established. Using *in vivo* passages of YFV in *Ae*. *albopictus*, we have selected viral strains presenting substitutions in NS1 gene. We did 10 passages of YFV-74018 on two distinct *Ae*. *albopictus* populations: (i) Manaus collected from a YFV-endemic area in Amazonia and (ii) PNMNI from a YFV-free area in the state of Rio de Janeiro. Full viral genomes were deep sequenced at each passage. We obtained two YFV strains presenting a non-synonymous substitution in the NS1 gene. Interestingly, they intervened at two different positions in NS1 gene according to the mosquito population: I2772T in *Ae*. *albopictus* Manaus and S3303N in *Ae*. *albopictus* PNMNI. Both substitutions reached fixation at the passage 10. Our data suggest that YFV has the potential for adaption to *Ae*. *albopictus* thereby posing a threat to most cities in South America where this mosquito is present.

## Introduction

In early January 2018, a mass immunization campaign was implemented to prevent the arrival and re-urbanization of Yellow fever (YF) in the populated cities of Rio de Janeiro and São Paulo states in Southeastern Brazil, which were declared free of YF for more than 70 years until 2017^1^ (http://portalms.saude.gov.br/noticias/agencia-saude/42655-febre-amarela-ministerio-da-saude-atualiza-casos-no-pais). According to Brazil’s Ministry of Health, between July 2017 and February 2018, 723 human cases were confirmed in the country, of which 237 resulted in death (http://portalms.saude.gov.br/noticias/agencia-saude/42655-febre-amarela-ministerio-da-saude-atualiza-casos-no-pais). A unique molecular signature was detected in the genome of YFV strains circulating in the current outbreak^2,3^.

Yellow fever virus (YFV), an arbovirus of the *Flavivirus* genus and the Flaviviridae family, causes a disease endemic to tropical regions of Africa and South America. It is a single-stranded, positive sense RNA virus with a genome of approximately 11 kb. Seven genotypes are described: 5 in Africa (2 in West Africa (WAI and WAII) and 3 in East/Central Africa (EA, ECA, and Angola)) and 2 in South America (SAI and SAII)^4^. YFV strains circulating in the Americas derived from the West African genotype and most isolates from Brazil mainly belong to the South American genotype I^5^. In spite of the availability of effective vaccines, YF remains an important public health problem in Africa and South America, with an annual incidence of around 200,000 cases and 30,000 deaths (http://www.who.int/emergencies/yellow-fever/en/); 90% of them occur in Africa and fatality rates often exceed 20% corresponding to severe forms characterized clinically by liver and kidney failure. In Africa, YFV circulates within three distinct cycles: (i) a jungle cycle where YFV is transmitted between non-human primates by canopy-dwelling mosquitoes such as *Aedes africanus*, (ii) an intermediate or savannah cycle involving several zoophilic mosquitoes, and (iii) an urban cycle where YFV is transmitted between humans by the domestic and human-biting mosquito *Aedes aegypti*. In the Americas, YFV only persists today in a jungle cycle between non-human primates and sylvatic mosquitoes. After its introduction following the slave trade, 300–400 years ago, YFV caused devastating outbreaks in American harbors and succeeded in establishing a sylvatic enzootic cycle within the Amazon, Araguaia, and Orinoco river basins^6,7^. The last documented *Aedes aegypti*-vectored epidemic occurred in 1928 and 1929 in the city of Rio de Janeiro, when 738 cases and 478 deaths were reported respectively^6^. Then, the Pan-American eradication program of *Ae*. *aegypti* led to an elimination of urban YF^8^. Brazil was certified free of *Ae*. *aegypti* in 1957^9^. Today, the jungle YFV cycle is still very active in Brazil, and it generates outbreaks every 6–10 years in the Southern, Southeast and Central-West regions, and every 14 years in Amazonia^10^. Humans are infected by the bite of forest canopy-dwelling mosquitoes of the genera *Haemagogus* (primary vectors; *H*. *janthinomys*, *H*. *leucocelaenus* and *H*. *albomaculatus*) and *Sabethes* (secondary vectors; *S*. *chloropteros*)^3,11,12^. However, with the increase of trade and travels with YF-endemic regions, imported cases are repeatedly reported outside YF historical regions; in March 2016, 11 Chinese workers returning from Angola developed disease symptoms^13^ in cities where competent *Aedes* mosquitoes were present. Thus, *Ae. aegypti* and *Aedes albopictus* may facilitate urban resurgence of YF in *Aedes*-infested regions of America^14^, Europe^15^ and Africa^16^.

In late 2016, a severe YFV epidemic was declared in southeastern Brazil, in a region highly infested by *Ae*. *aegypti* and *Ae. albopictus*^2,17^. *Ae*. *albopictus* was first detected in southeastern Brazil in 1986^18^. This opportunistic species is able to colonize distinct habitats acting as a possible link between the jungle cycle and the urban cycle^19,20^. Despite being susceptible to infection with YFV in laboratory conditions, infected *Ae*. *albopictus* have never been found in natural settings^17,19^. However, it is coincidently more densely distributed in the Southeast region where the YFV is actively transmitted^21^ (http://portalarquivos.saude.gov.br/images/pdf/2017/junho/02/COES-FEBRE-AMARELA–INFORME-43–Atualiza----o-em-31maio2017.pdf), enhancing significantly the chances of contacts between this mosquito and the virus. We hypothesize that YFV can be experimentally selected for a potential transmission by *Ae*. *albopictus*. We passaged an YFV isolate (SAI lineage 1D, isolated in 2001) on two distinct populations of *Ae*. *albopictus*, collected from a YFV-endemic area (Manaus) and a YFV-free area (Rio de Janeiro). Heads of 30 mosquitoes (containing disseminated virus from the midgut) were pooled at late days post-infection and inoculated for amplification on *Ae*. *albopictus* C6/36 cells. Then newly produced virions were harvested and used for the next mosquito oral infection. After five rounds of cycling on *Ae*. *albopictus*, virus was detected in mosquito saliva ready to be transmitted by bite. After five additional passages using virus collected from saliva, resulting viral strains were examined to identify genetic changes in the viral genome.

## Results

### YFV is excreted in mosquito saliva after 4 passages in *Ae*. *albopictus*

**Figure 1:**
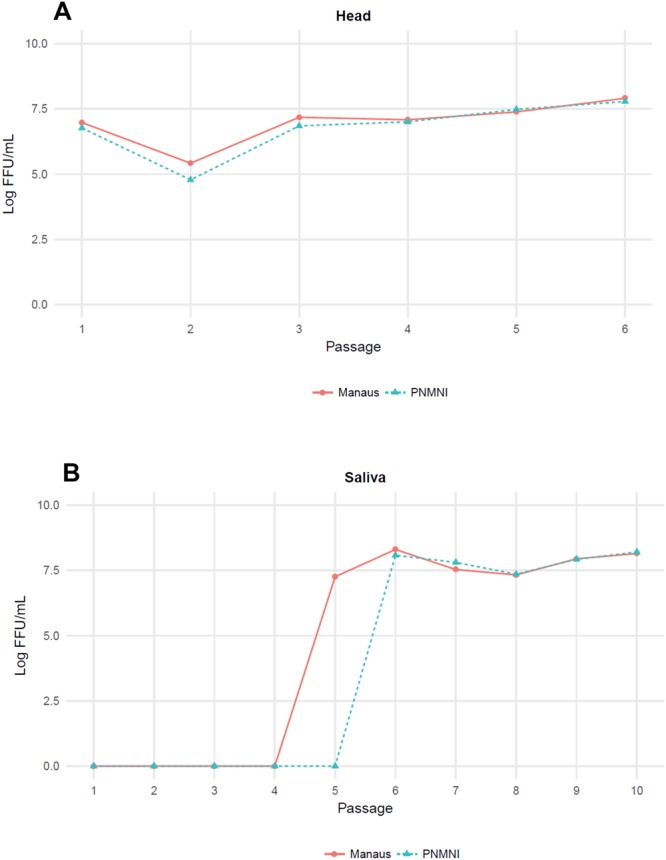
Viral titers obtained at each passage of YFV on *Ae*. *albopictus* mosquitoes (**A**) using virus collected from head and (**B**) from saliva. Mosquitoes were orally infected with YFV-74018. After 21 days, mosquitoes were processed as follows: (i) heads were collected and ground, and (ii) mosquito saliva were pooled. Head and saliva homogenates were incubated on *Ae*. *albopictus* C6/36 cells for 8 days. Collected supernatants were used to initiate the next passage. 30 mosquitoes were used at each passage.

After a first blood meal at a titer of 10^6.5^ FFU/mL, 30 mosquitoes were examined at 21 days post-infection (dpi) and no viral particles were detected in any mosquito saliva. Then mosquito heads which most likely contain virus that has disseminated from the midgut into the hemocele, were ground and amplified once on C6/36 cells. Passages 1 to 4 were performed as described previously (Fig. 1A); except P2, viral titers obtained after incubation of mosquito head homogenates on C6/36 cells were around 10^7^ FFU/mL. The virus became detectable in mosquito saliva from P5 (Fig. 1B). Then, next passages (P6-S to P10-S) were performed using virus produced from saliva pooled from 30 mosquitoes. Viral titers in saliva remained high, fluctuating from 10^8–8.3^ FFU/mL (P6) to 10^8.1-8.2^ FFU/mL (P10) (Fig. 1B).

### Experimental selection of YFV transmitted by *Ae*. *albopictus*

**Figure 2:**
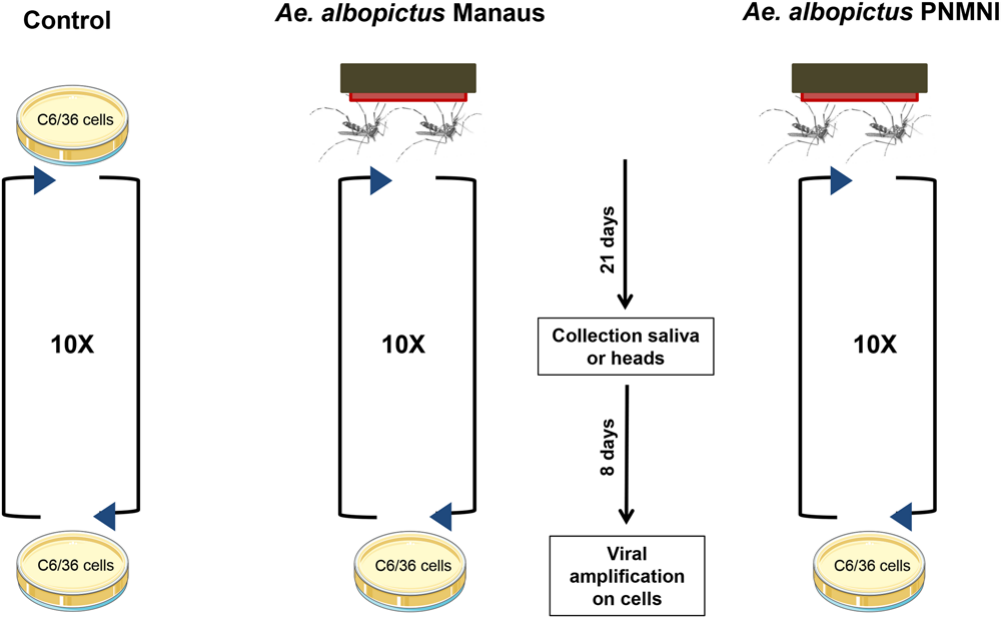
Experimental design to enhance YFV transmission by *Ae*. *albopictus*. The YFV-74018 was passaged 10 times on two populations of *Ae*. *albopictus*: (i) Manaus collected in a YFV-endemic area in Amazonia, and (ii) PNMNI collected from Rio de Janeiro, a YFV-free area. Each passage includes: the oral infection of mosquitoes with YFV, collection of mosquito saliva at day 21 post-infection, viral amplification on *Ae*. *albopictus* derived C6/36 cell culture, and initiation of the next passage using the viral suspension obtained. Control isolates were serially passaged 10 times on C6/36 cells.

To test whether YFV can become adapted for a potential transmission by *Ae*. *albopictus*, YFV-74018 was submitted to 10 passages in two populations of *Ae*. *albopictus* collected from two distinct regions: (i) Manaus in a YFV-endemic area and (ii) PNMNI in a YFV-free area of Rio de Janeiro (Fig. 2). Additionally, the virus was passaged 10 times in duplicate in *Ae*. *albopictus* C6/36 cells as a cell culture control. Full viral genomes were examined by deep sequencing at each passage (1–10) and for passages 0 (parental strain), and 10 for the C6/36 cells control.

**Figure 3:**
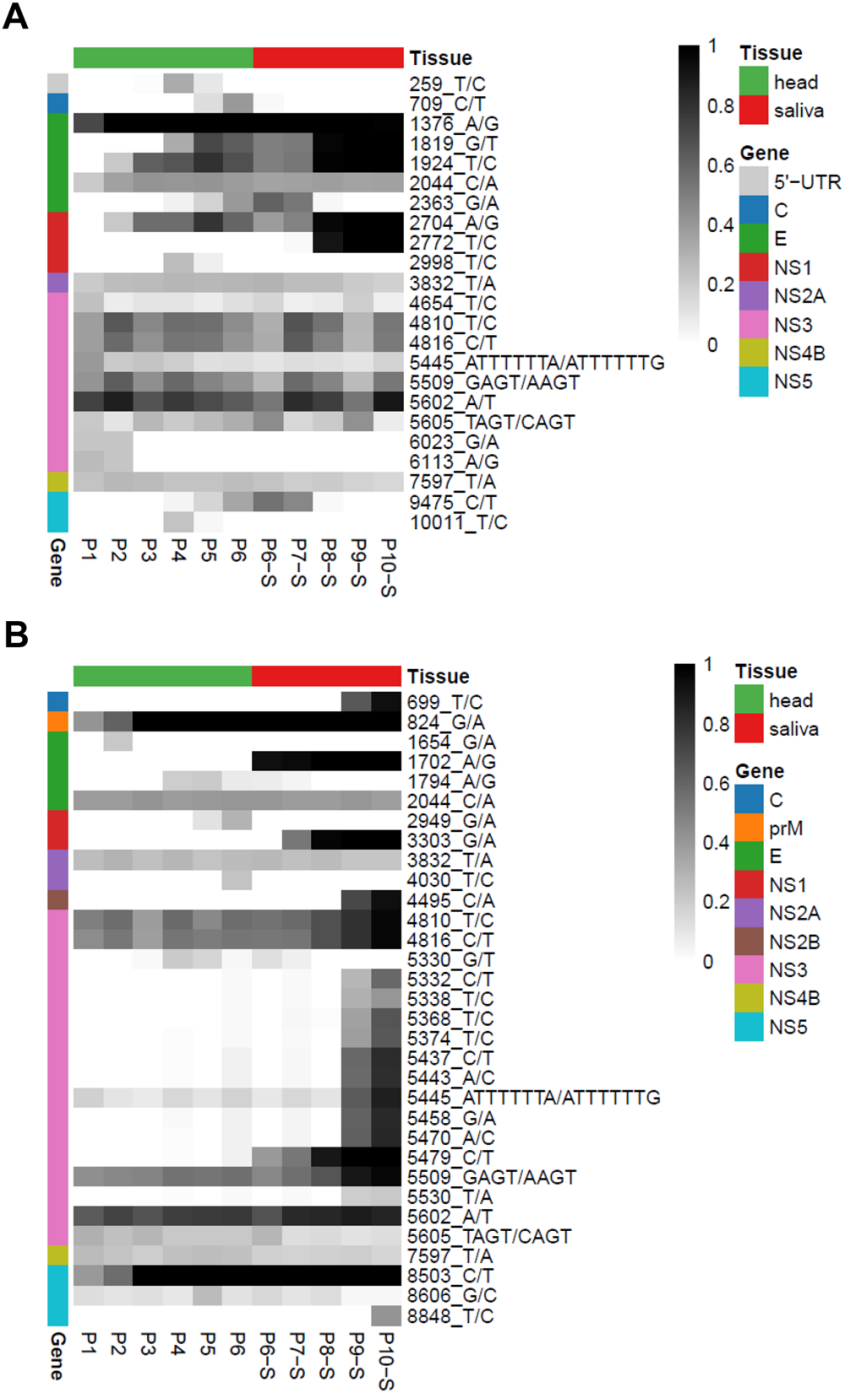
Heatmap indicating variant frequency differences between the reference genome YFV-74018 and isolates selected after passages on *Ae*. *albopictus* Manaus (**A**) and PNMNI (**B**). All 10 passages were deep sequenced: P1 to P5 were performed using homogenates of mosquito heads, and P6-S to P10-S with mosquito saliva. The intensity of black corresponds to the variant frequency. Only variants for which the frequency is above 20% are shown.

The YFV-74018 yielded a mean sequencing depth of 424X covering 97.3% of the reference genome at >100X. All passages had a mean coverage between 352X and 494X, paving between 85.6 and 98.6% of the reference genome at >100X. When YFV-74018 was serially passaged on the C6/36 cells control, no major changes in single nucleotide variants (SNV) frequencies were detected. Contrariwise, consensus level variants were detected when YFV-74018 was passaged in *Ae*. *albopictus* mosquitoes, Manaus (Fig. 3A) and PNMNI (Fig. 3B). In Manaus mosquitoes, a total of 23 consensus level variants were detected from P1 to P10-S: 22 from P1 to P6 when using mosquito head homogenates as source for the next passage (head-derived) and 16 variants from P6-S to P10-S when pools of mosquito saliva were used for passages (saliva-derived) (Fig. 3A). Sixteen among 23 were located in non-structural genes (NS1, NS2A, NS3, NS4B and NS5). A group of 10 consensus level variants (positions 1376, 2044, 3832, 4810, 4816, 5445, 5509, 5602, 5605, and 7597) were present in all passages (head- or saliva-derived). A group of 6 consensus level variants were specific to head-derived passages (positions 259, 709, 2998, 6023, 6113, and 10011) and one to saliva-derived passages (position 2772). Interestingly, this last consensus level variant (position 2772) was first detected at P8-S and become fixed at P10-S; it is located in the NS1 gene (Fig. 3A).

In PNMNI mosquitoes, a total of 32 consensus level variants were detected from P0 to P10-S: 26 from P1 to P6 and 29 variants from P6-S to P10-S (Fig. 3B). Twenty-six among 32 were located in non-structural genes (NS1, NS2A, NS2B, NS3, NS4B and NS5). A group of 11 consensus level variants (positions 824, 2044, 3832, 4810, 4816, 5509, 5602, 5605, 7597, 8503, and 8606) were present in all passages (head- or saliva-derived). Three consensus level variants were specific to head-derived passages (positions 1654, 2949, and 4030) and 6 to saliva-derived passages (positions 699, 1702, 3303, 4495, 5530, and 8848). Two consensus level variants (positions 1702 and 3303) were first detected in saliva-derived passages and became fixed at P10-S; the position 1702 was located in E gene and 3303 in NS1 gene (Fig. 3B).

### Emergence of YFV variants in mosquito saliva

When focusing on the viral populations present in mosquito saliva from P6-S (as in previous passages, no virus was found in saliva), we detected three consensus level variants: A1702G, T2772C, and G3303A, reaching the 100% fixation at P10-S.

**Figure 4:**
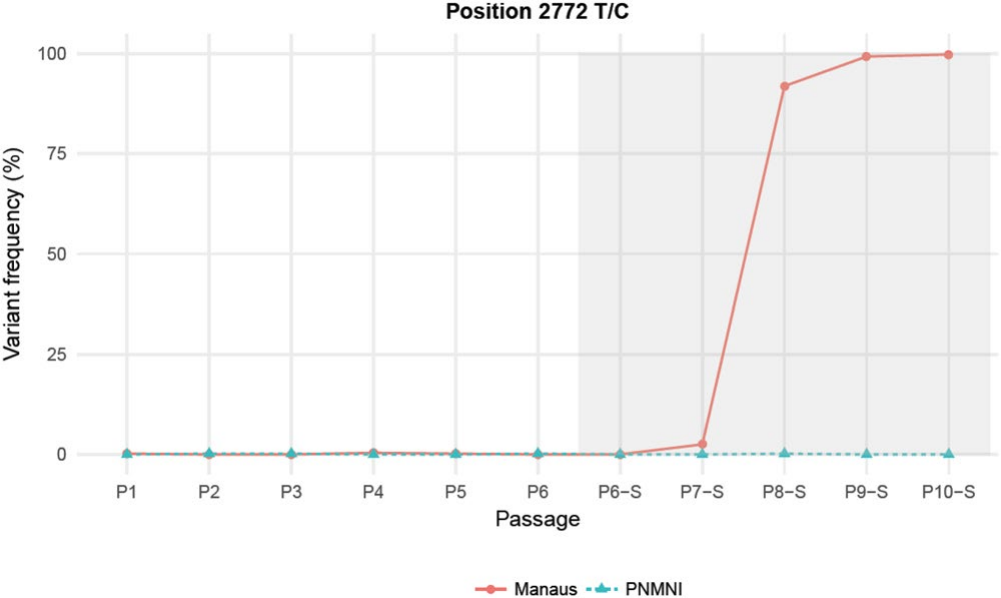
Frequency of the T2772C variant detected after the passage P7-S in *Ae*. *albopictus* Manaus. This substitution corresponds to a non-synonymous change from isoleucine to threonine in NS1 gene.

**Figure 5:**
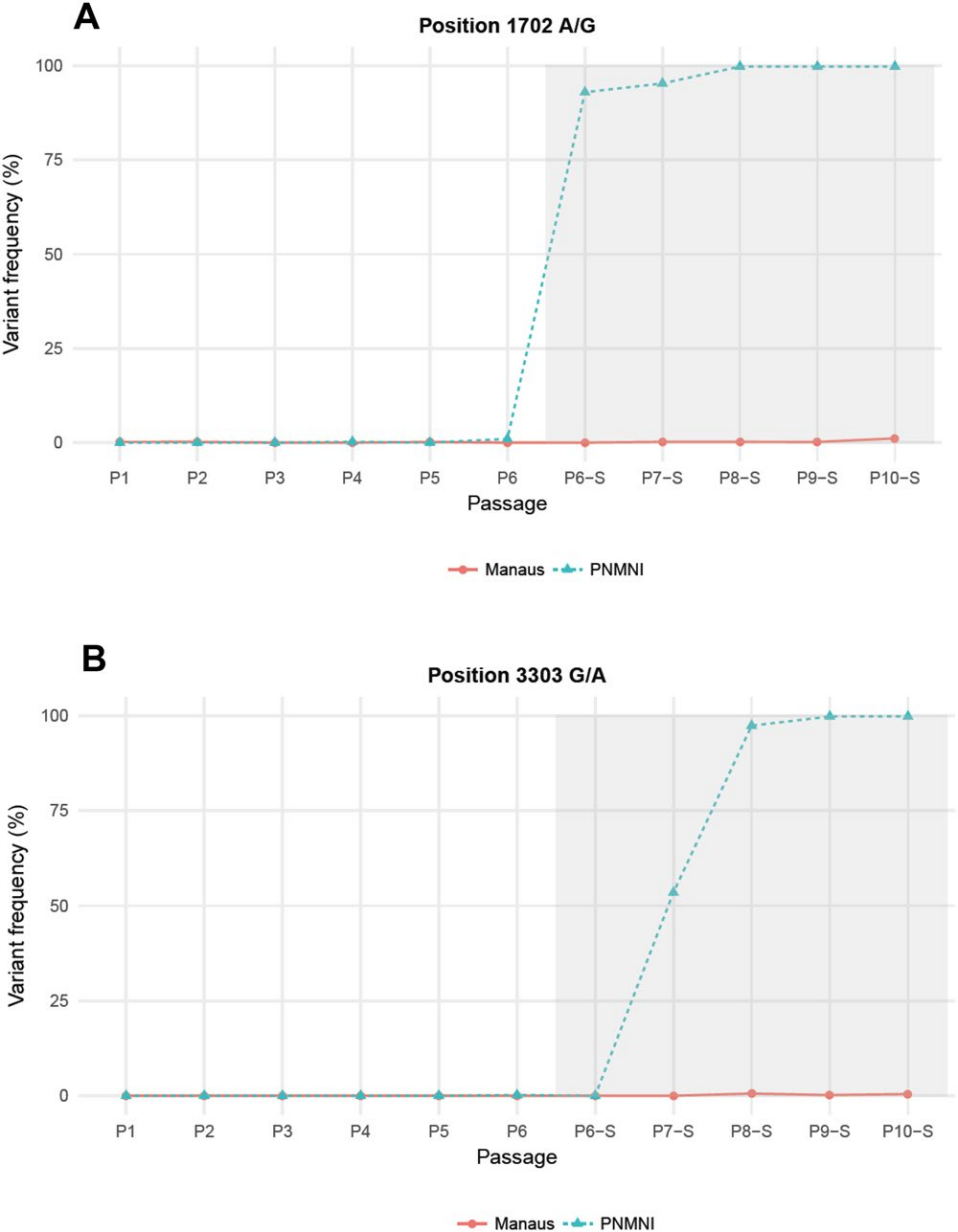
Frequencies of the K1702K and S3303N variants detected after the passages P6-S and P7-S respectively in *Ae*. *albopictus* PNMNI. Only the substitution S3303N corresponds to a non-synonymous change from serine to asparagine in NS1 gene.

In *Ae*. *albopictus* Manaus, one variant with T2772C in NS1 gene detected from P8-S corresponded to a non-synonymous substitution from isoleucine to threonine. It started to be detected at P7-S (2.5%) and increased rapidly to reach fixation in three passages at P10-S (Fig. 4). In *Ae*. *albopictus* PNMNI, two variants were detected: A1702G from P6-S and G3303A from P7-S (Fig. 5). The A1702G change in E gene led to synonymous substitution (lysine), reached 80% of frequency from P6-S and became fixed at P10-S (frequency = 100%) (Fig. 5A). The G3303A change in NS1 gene induced a non-synonymous substitution from serine to asparagine and reached 100% from P8-S (Fig. 5B). These results indicate that YFV-74018 can accumulate mutations that facilitate virus transmission after passages on *Ae*. *albopictus*. However, passaging on Manaus or PNMNI did not select the same substitution.

## Discussion

Here we describe YFV strains selected after 10 passages on *Ae*. *albopictus* mosquitoes to mimic repeated interactions of the virus with an invasive mosquito previously described as a poor YFV vector. Against all expectations, YFV has been detected in field-collected *Ae*. *albopictus* in Southeastern Brazil in 2017 (http://www.iec.gov.br/portal/descoberta/). YFV variants selected after 10 passages differed according to the mosquito population: Manaus mosquitoes selected one variant T2772C inducing a non-synonymous change in NS1 while PNMNI selected two variants, A1702G leading to a synonymous substitution in E gene and G3303A to a non-synonymous substitution in NS1 gene.

*Ae*. *albopictus* has not been found infected with YFV in South America until recently^17^. Alarmingly, YFV was detected in field-collected *Ae*. *albopictus* in Southeastern Brazil in 2017 (http://www.iec.gov.br/portal/descoberta/). This mosquito originally from Southeast Asia^22^, was firstly found in Brazil in the state of Rio de Janeiro in 1986^18^ and in Manaus, state of Amazonas, in 2002^23^. It colonizes a wide range of habitats from peri-urban sites to forested environments and thus comes into close contacts with the YFV jungle cycle where the virus persists despite the mass Pan-American program of YF control during the first half of the 20^th^ century^24^. *Ae*. *albopictus* from Manaus are likely genetically different from *Ae*. *albopictus* from Rio de Janeiro owing to differences on date and sources of introduction^25^ suggesting that both *Ae*. *albopictus* populations behave as different filters for selecting viral variants^26^.

A high number of consensus level variants were detected from P1 to P10-S: 32 in PNMNI and 23 in Manaus. Genetic characteristics of viruses passaged on mosquitoes can be described using mean of nonsynonymous to synonymous nucleotide substitutions (*dN*/*dS*) per site, codon usage bias and frequencies of CG. *dN*/*dS* (0.45–0.67; Supplementary Table 1) were 10–15 times higher than values obtained from field-isolated YFV strains (0.043^27^; <0.2^5^). It suggests that our experimental design by forcing adaptation of YFV to *Ae*. *albopictus* produced a high purifying selection pressure generating a vast majority of synonymous mutations. In addition, only slight variations of codon usage bias were detected with values close to 53 suggesting a random codon usage for each amino acid (Supplementary Table 2). Moreover, the number of CG dinucleotides between passage 1 and passage 10 was slightly different for *Ae*. *albopictus* PNMNI and comparable for *Ae*. *albopictus* Manaus suggesting a low rate of evolution without likely any phenotypic effects (Supplementary Table 3).

Interestingly, beside variants present all along the 10 passages, 7 were detected only in mosquito saliva from passage 6: one in *Ae*. *albopictus* Manaus and 6 in *Ae*. *albopictus* PNMNI. Among them, three reached fixation at passage 10: I2772T in *Ae*. *albopictus* Manaus and two others in *Ae*. *albopictus* PNMNI (K1702K and S3303N). The synonymous 1702 substitution in E gene would not cause any significant change. On the other hand, the two other substitutions I2772T and S3303N are located in the NS1 gene. Genetic characteristics of viruses passaged on *Ae*. *albopictus* can be described using codon usage bias and frequencies of CG. NS1 is a highly conserved non-structural protein which has been described under different forms including a secreted hexamer protein. NS1 has been incriminated in eliciting the immune response^28^, activating the TLRs and inhibiting the complement system^29,30^. Disease severity and increased viremia are likely correlated to a high concentration of NS1^31^. Mutations in the NS1 gene may modify its ability to trigger immune responses and avoid being the target of antivirals. The detection of these substitutions in experimental conditions with an increase of virus titers in *Ae*. *albopictus* saliva should alert us about the potential of YFV to emerge from a sylvatic cycle to reach peri-urban areas where this mosquito is well established. Collectively, this may facilitate the establishment of urban YF cycles in countries where *Ae*. *albopictus* is present.

## Methods

### Ethics Statements

The Institut Pasteur animal facility has received accreditation from the French Ministry of Agriculture to perform experiments on live animals in compliance with the French and European regulations on care and protection of laboratory animals. This study was approved by the Institutional Animal Care and Use Committee (IACUC) at the Institut Pasteur. Mosquito collections in Brazil were approved by local environmental authorities (PNMNI license 001/14–15; SISBIO-MMA licenses 37362-2 and 012/2016). No specific permits were required for performing mosquito collections in Brazil. This study did not involve endangered or protected species.

### Viruses

Mosquitoes were orally infected with one YFV isolate belonging to the SAI genotype, isolated from a human fatal case in 2001; it corresponds to the lineage 1D FIOCRUZ 74018/MG/01 (YFV-74018)^32^. The isolate has been passaged four times on *Ae*. *albopictus* C6/36 cells and viral stocks were stored at −80 °C until use for mosquito challenges.

### Mosquitoes

Two populations of *Ae*. *albopictus* were originated from a YFV-endemic area in the Amazon (Manaus) and a YFV-free area in the state of Rio de Janeiro (Parque Nacional Municipal de Nova Iguaçu-PNMNI)^17^. Generation F1 for Manaus and PNMNI respectively were challenged with YFV-74018. Eggs were immerged in dechlorinated tap water and larvae were fed with yeast tablets renewed every 2–3 days. Pupae were collected manually and grouped in bowls placed in cages. Adults were fed ad libitum with a 10% sucrose solution in standardized conditions (27 ± 1 °C; 80 ± 10% RH; 16 h:8 h light:dark cycle).

#### Experimental selection by serial passages on *Ae*. *albopictus*

For the first passage, mosquitoes were orally challenged with YFV-74018 provided in a blood-meal (washed rabbit erythrocytes) at a final titer of 10^6.5^ FFU/mL as previously described^17^. Engorged mosquitoes were incubated at 28 °C for 21 days and then processed for saliva collection^33^. Saliva or head homogenates of 30 mosquitoes were pooled and the volume of the pool was adjusted to 600 µL with L15 prior to filtration through a Millipore H membrane (0.22 µm). An aliquot of 300 µL of each sample was used to inoculate a sub-confluent flask (25 cm^2^) of C6/36 *Ae*. *albopictus* cells. After 1 hour, the inoculum was discarded and cells were rinsed once with medium. L15 medium (5 mL) complemented with 2% FBS were added and cells were incubated for 8 days at 28 °C. Cell culture supernatants were then collected and provided to mosquitoes to run the next passage. Passages 1 to 5 were performed using homogenates of mosquito heads as saliva was not infectious or at a very scanty viral titer, and passages 6 to 10 with mosquito saliva which became infectious from P5. C6/36 supernatants collected at each passage were used undiluted for the next mosquito blood-meal without titration. Control isolates corresponded to serially passaged viruses on C6/36 cells to identify mutations resulting from genetic drift or adaptation to insect cell line.

#### Viral titration by focus forming assay

Samples were titrated by focus fluorescent assay on *Ae*. *albopictus* C6/36 cells^34^. Samples were serially diluted and inoculated onto C6/36 cells in 96-well plates. After an incubation of 5 days at 28 °C, cells were stained using hyper-immune ascetic fluid specific to each virus as the primary antibody and conjugated goat anti-mouse as the secondary antibody. Titers were expressed as FFU/mL.

#### Virus deep sequencing

Total RNA was extracted from cell culture supernatant using the Nucleospin RNA II kit (Macherey-Nagel, Hœrdt, France) according to the manufacturer’s instructions. Six overlapping amplicons were produced using the reverse transcriptase Platinum® Taq High Fidelity polymerase enzyme (Thermo Fisher Scientific, Massachusetts, USA) and specific primers (Supplementary Table 4). PCR products were pooled in equimolar proportions. After Qubit quantification using Qubit® dsDNA HS Assay Kit and Qubit 2.0 fluorometer (ThermoFisher Scientific), amplicons were fragmented (sonication) into fragments of 200 bp long. Libraries were built adding barcode, for sample identification, and primers to fragmented DNA using AB Library Builder System (ThermoFisher Scientific). To pool equimolarly the barcoded samples, a quantification step by the 2100 Bioanalyzer instrument (Agilent Technologies, California, USA) was performed. An emulsion PCR of the pools and loading on a 520 chip were realised using the automated Ion Chef instrument (ThermoFisher Scientific). Sequencing was performed using the S5 Ion torrent technology (ThermoFisher Scientific) following manufacturer’s instructions. Consensus sequence was obtained after mapping the reads on reference (inoculum strain) using CLC genomics workbench software (Qiagen, Hilden, Germany). A *de novo* contig was also produced to ensure that the consensus sequence was not affected by the reference sequence.

### Bioinformatic analysis

#### Quality control of the data

The quality control of the sequencing data has been performed using FastQC (v 0.11.5) (https://www.bioinformatics.babraham.ac.uk/projects/fastqc/) and MultiQC (v 0.7)^35^. Reads were trimmed using Trimmomatic (v 0.36)^36^ with the parameters “Leading:2 Trailing:3 Slidingwindow:4:15 Minlen:36”. In order to filter any reads which could come from the mosquito host, sequences were mapped using BWA mem (v 0.7.7)^37^ against *Ae*. *albopictus* derived C6/36 cells (assembly GCA001876365.2). The SAM file produced was converted, sorted and indexed using Samtools (v 1.3)^38^ and the unmapped reads were extracted using SamToFastq from Picard Tools (v 2.8.1) (http://broadinstitute.github.io/picard/).

#### Variant calling

These unmapped reads were then mapped using BWA mem (v 0.7.7)^37^ and default parameters against the reference genome YFV-74018^32^. The duplicated reads were marked with MarkDuplicates from Picard Tools (v 2.8.1) (http://broadinstitute.github.io/picard/). Finally, the variant calling was done with freebayes (v 1.1.0)^39^ using the option -pooled-continuous. A filter has been applied with vcffilter from vcflib (v 1.0.0)^39^ to select only SNP variants with a quality score above 20. The results analysis has finally been done under R (v 3.3.1) (https://www.R-project.org) with the packages VariantAnnotation (v 1.20.3)^40^, pheatmap (v 1.0.8) (https://CRAN.R-project.org/package=pheatmap) and ggplot2 (v2.2.1) (https://www.springer.com/us/book/9780387981413).

## Electronic supplementary material

Supplementary Table 1–4
