# Supplementary material for: Experimental Adaptation of the Yellow Fever Virus to the Mosquito Aedes albopictus and Potential risk of urban epidemics in Brazil, South America
Source: Sci Rep. 2018 Sep 25;8:14337. doi: 10.1038/s41598-018-32198-4 (PMC6156417; doi:10.1038/s41598-018-32198-4)
Supplement: Supplementary file 1 — Supplementary Table 1–4 [file 41598_2018_32198_MOESM1_ESM.pdf]

Experimental Adaptation of the Yellow Fever Virus to the Mosquito *Aedes albopictus* and Potential risk of urban epidemics in Brazil, South America. Fadila Amraoui,[1] Adrien Pain,[2,§] Géraldine Piorkowski,[3,4,§] Marie Vazeille,[1] Dinair Couto-Lima,[5] Xavier de Lamballerie,[3,4,#] Ricardo Lourenço-de-Oliveira,[5,#] Anna-Bella Failloux[1,#]

Supplementary Table 1 | Nucleotide substitutions in YFV at different passages on *Ae. albopictus* mosquitoes (Manaus and PNMNI). The mean ratio of nonsynonymous to synonymous nucleotide substitutions (*dN*/*dS*) per site was calculated. YFV-74018 was used as the reference genome.

Viral strain		Non-Synonymous	Synonymous	*dN/dS*
YFV-74018				
PNMNI.P1		14	31	0.45
PNMNI.P2		13	29	0.45
PNMNI.P3		13	31	0.42
PNMNI.P4		13	28	0.46
PNMNI.P5		13	29	0.45
PNMNI.P6		13	28	0.46
PNMNI.P6-S		13	29	0.45
PNMNI.P7-S		12	28	0.43
PNMNI.P8-S		12	28	0.43
PNMNI.P9-S		12	21	0.57
PNMNI.P10-S		12	18	0.67
Manaus.P1		13	31	0.42
Manaus.P2		13	29	0.45
Manaus.P3		13	30	0.43
Manaus.P4		13	27	0.48
Manaus.P5		13	27	0.48
Manaus.P6		13	29	0.45
Manaus.P6-S		12	30	0.40
Manaus.P7-S		12	27	0.44
Manaus,P8-S		12	27	0.44
Manaus.P9-S		12	29	0.41
Manaus.P10-S		12	26	0.46

P, Passage; S, Saliva.

Supplementary Table 2 | Codon usage bias in YFV at different passages on *Ae. albopictus* mosquitoes (Manaus and PNMNI). It was measured using the effective number of codon prime (Nc') [1], giving values ranging from 20 (only one codon used for each amino acid) to 61 (random codon usage for each amino acid).

Viral strain		Nc'
YFV-74018		53.03
PNMNI.P1		52.88
PNMNI.P2		52.86
PNMNI.P3		52.86
PNMNI.P4		52.86
PNMNI.P5		52.86
PNMNI.P6		52.86
PNMNI.P6-S		52.87
PNMNI.P7-S		52.88
PNMNI.P8-S		52.88
PNMNI.P9-S		52.95
PNMNI.P10-S		52.94
Manaus.P1		52.87
Manaus.P2		52.87
Manaus.P3		52.90
Manaus.P4		52.90
Manaus.P5		52.92
Manaus.P6		52.92
Manaus.P6-S		52.88
Manaus.P7-S		52.89
Manaus.P8-S		52.94
Manaus.P9-S		52.94
Manaus.P10-S		52.94

P, Passage; S, Saliva.

Supplementary Table 3 | Number of CG dinucleotides in YFV at different passages on *Ae. albopictus* mosquitoes (Manaus and PNMNI). It corresponds to the quotient of the probability of finding a dinucleotide at a given sequence divided by the product of the probabilities of finding each nucleotide forming the dinucleotide in the same sequence. It was calculated using the Dambe software [2].

Viral strain		CG (%)
YFV-74018		13.69
PNMNI.P1		13.40
PNMNI.P2		13.52
PNMNI.P3		13.46
PNMNI.P4		13.52
PNMNI.P5		13.52
PNMNI.P6		13.52
PNMNI.P6-S		13.52
PNMNI.P7-S		13.52
PNMNI.P8-S		13.52
PNMNI.P9-S		13.63
PNMNI.P10-S		13.63
Manaus.P1		13.40
Manaus.P2		13.46
Manaus.P3		13.34
Manaus.P4		13.40
Manaus.P5		13.46
Manaus.P6		13.40
Manaus.P6-S		13.34
Manaus.P7-S		13.46
Manaus,P8-S		13.40
Manaus.P9-S		13.40
Manaus.P10-S		13.46

P, Passage; S, Saliva.

Supplementary Table 4 | Set of primers used for the sequencing of YFV strains.

Sequence (5' to 3')	Forward/Reverse	nt position
ATGTCTGGTCGTAAAGCTCAG	Forward 1	119
CTTCCCTCTTTGTGCCACTG	Reverse 1	2154
CCTCAACCAATGATGATGAAG	Forward 2	2040
CACCTCAGCCATAGTGACAG	Reverse 2	3997
GCAGCCATGGTGGAGATTG	Forward 3	3832
AACTTCCATGAGCCACCATAGG	Reverse 3	4711
GATGGGATTTATGGCATATTCCAGTC	Forward 4	4636
GCTCRCTGGGTATGTCCGTt	Reverse 4	5595
GCTAGGGCAAATGAAAGyGCA	Forward 5	5494
AGCCACGyTCATGGAACCA	Reverse 5	7832
GTGGGAGTCATGTACAATCTA	Forward 6	7592
TCAGATAAGCTCACCCGGTT	Reverse 6	10354

References

1 Novembre, J. A. Accounting for background nucleotide composition when measuring codon usage bias. *Mol Biol Evol* **19**, 1390-1394, doi:10.1093/oxfordjournals.molbev.a004201 (2002).

2 Xia, X. & Xie, Z. DAMBE: software package for data analysis in molecular biology and evolution. *J Hered* **92**, 371-373 (2001).
